# Supplementary material for: RNA-binding proteins hnRNPM and ELAVL1 promote type-I interferon induction downstream of the nucleic acid sensors cGAS and RIG-I
Source: EMBO J. 2024 Dec 20;44(3):824–53. doi: 10.1038/s44318-024-00331-x (PMC11791083; doi:10.1038/s44318-024-00331-x)
Supplement: Supplementary file 1 — Appendix [file 44318_2024_331_MOESM1_ESM.pdf]

Appendix

RNA-binding proteins hnRNPM and ELAVL1 promote type-I interferon induction downstream of the nucleic acid sensors cGAS and RIG-I

Table of Contents

Appendix Figure S1: MS-444 is tolerated well by THP-1 cells and primary human fibroblasts.....2

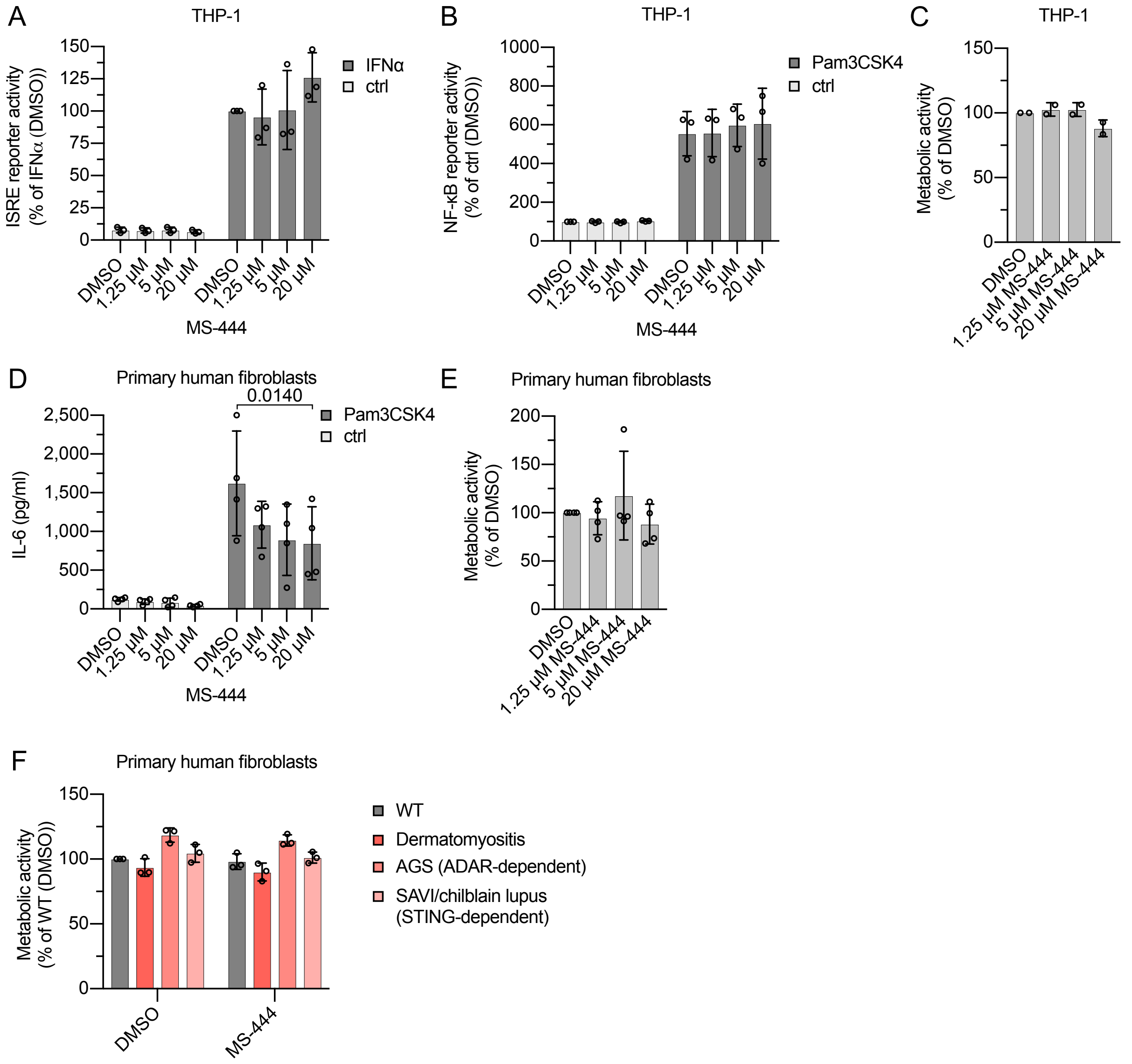

Appendix Figure S1: MS-444 is tolerated well by THP-1 cells and primary human fibroblasts.

(A) THP-1 cells were pre-treated with MS-444/DMSO for 2 h and then stimulated with IFNα (5000 U/ml) or left non-stimulated (ctrl) in MS-444/DMSO-containing medium. After 16 h, ISRE reporter activation was determined.

(B) THP-1 cells were pre-treated with MS-444/DMSO for 2 h and then stimulated with Pam3CSK4 (0.5 µg/ml) or left non-stimulated (ctrl) in MS-444/DMSO-containing medium. After 16 h, NF-κB reporter activation was determined.

(C) MTT assay of the cells depicted in (A) in the non-stimulated condition (mean ± SD of biological replicates).

(D) Primary human fibroblasts were pre-treated with MS-444/DMSO for 2 h and then stimulated with Pam3CSK4 (0.5 µg/ml) or left non-stimulated (ctrl) in MS-444/DMSO-containing medium. After 16 h, IL-6 secretion was analyzed. ctrl, non-stimulated.

(E) MTT assay of the cells depicted in (D) in the non-stimulated condition.

(F) MTT assay of primary human fibroblasts from a healthy donor (WT) or from patients with dermatomyositis, AGS, or SAVI/chilblain lupus treated for 16 h with 20 µM MS-444 or DMSO

For (A), (B): mean ± SD of biological replicates, two-way ANOVA, Dunnett's multiple comparisons test.

For (D), (F): mean ± SD of biological replicates, two-way ANOVA, Šídák's multiple comparisons test.

For (C), (E): mean ± SD of biological replicates, one-way ANOVA, Dunnett's multiple comparisons test.
