# Supplementary material for: RNA-binding proteins hnRNPM and ELAVL1 promote type-I interferon induction downstream of the nucleic acid sensors cGAS and RIG-I
Source: EMBO J. 2024 Dec 20;44(3):824–53. doi: 10.1038/s44318-024-00331-x (PMC11791083; doi:10.1038/s44318-024-00331-x)
Supplement: Supplementary file 11 — Source data Fig. 1 [file 44318_2024_331_MOESM11_ESM.zip › SD figure 1/1I/1I.pdf]

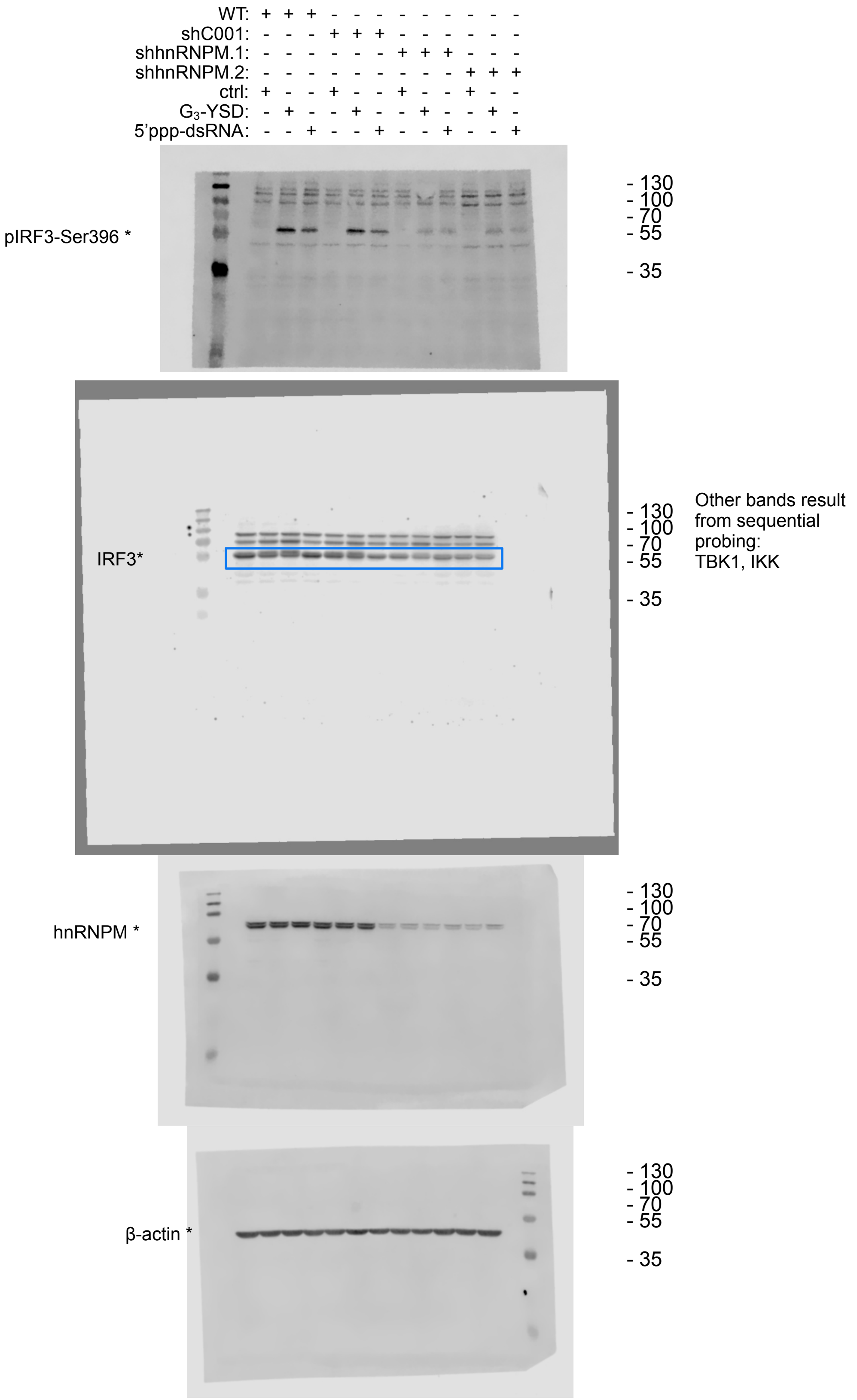
WT: + + + - - - - - - - - -
shC001: - - - + + + - - - - - -
shhnRNPM.1: - - - - - - + + + - - -
shhnRNPM.2: - - - - - - - - - + + +
ctrl: + - - + - - + - - + - -
G3-YSD: - + - - + - - + - - + -
5’ppp-dsRNA: - - + - - + - - + - - +
pIRF3-Ser396 *
- 130
- 100
- 70
- 55
- 35
IRF3*
- 130
- 100
- 70
- 55
- 35
Other bands result from sequential probing: TBK1, IKK
hnRNPM *
- 130
- 100
- 70
- 55
- 35
β-actin *
- 130
- 100
- 70
- 55
- 35
