# Supplementary figures and images for: RNA-binding proteins hnRNPM and ELAVL1 promote type-I interferon induction downstream of the nucleic acid sensors cGAS and RIG-I
Source: EMBO J. 2024 Dec 20;44(3):824–53. doi: 10.1038/s44318-024-00331-x (PMC11791083; doi:10.1038/s44318-024-00331-x)

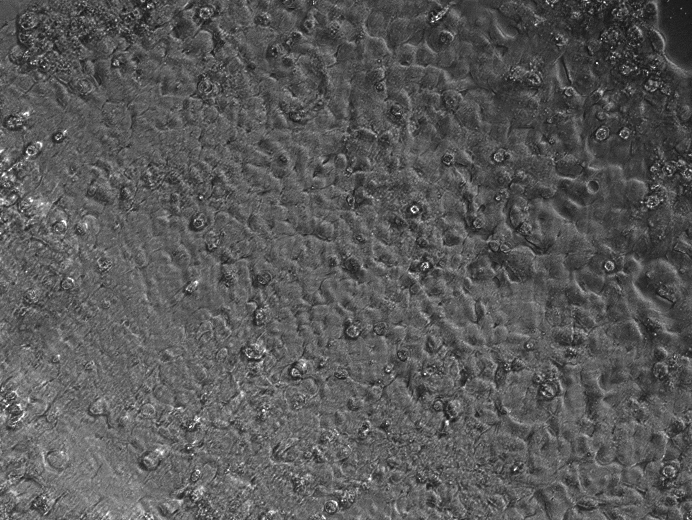

Supplement: Supplementary file 11 — Source data Fig. 1 [file 44318_2024_331_MOESM11_ESM.zip › SD figure 1/1A/hnRNPM-GFP/Aufnahme-3008-hnRNPM-GFP CVB3-FLAG trans.tif]

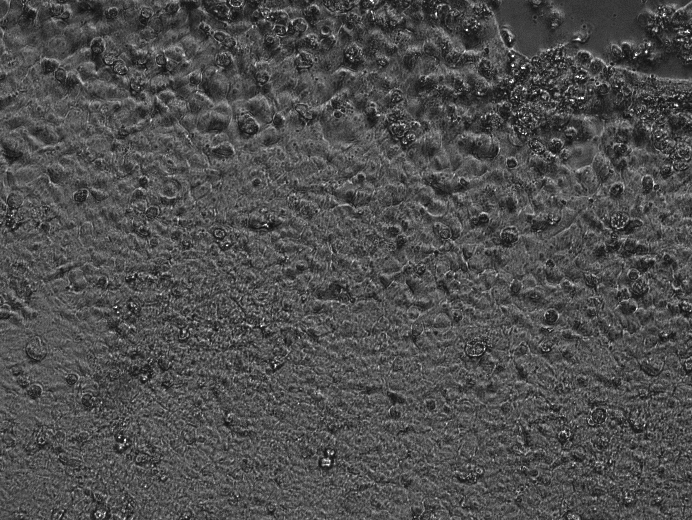

Supplement: Supplementary file 11 — Source data Fig. 1 [file 44318_2024_331_MOESM11_ESM.zip › SD figure 1/1A/hnRNPM-GFP/Aufnahme-3008-hnRNPM-GFP PV-FLAG trans.tif]

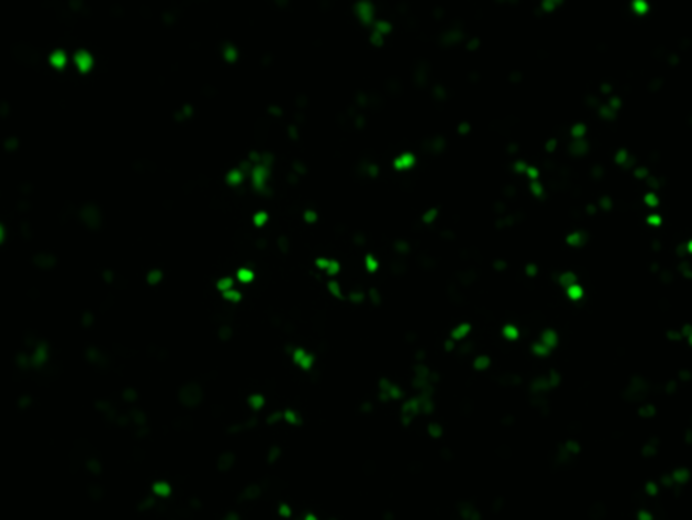

Supplement: Supplementary file 11 — Source data Fig. 1 [file 44318_2024_331_MOESM11_ESM.zip › SD figure 1/1A/hnRNPM-GFP/Aufnahme-3008-hnRNPM-GFP +pBluescript.tif]

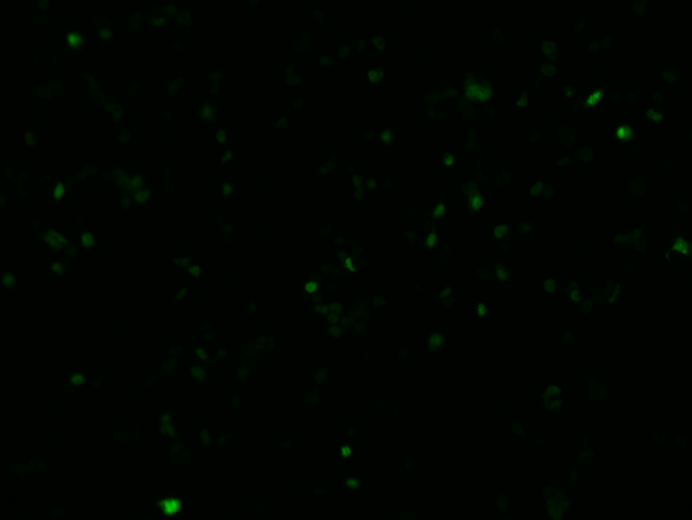

Supplement: Supplementary file 11 — Source data Fig. 1 [file 44318_2024_331_MOESM11_ESM.zip › SD figure 1/1A/hnRNPM-GFP/Aufnahme-3008-hnRNPM-GFP CVB3-FLAG.tif]

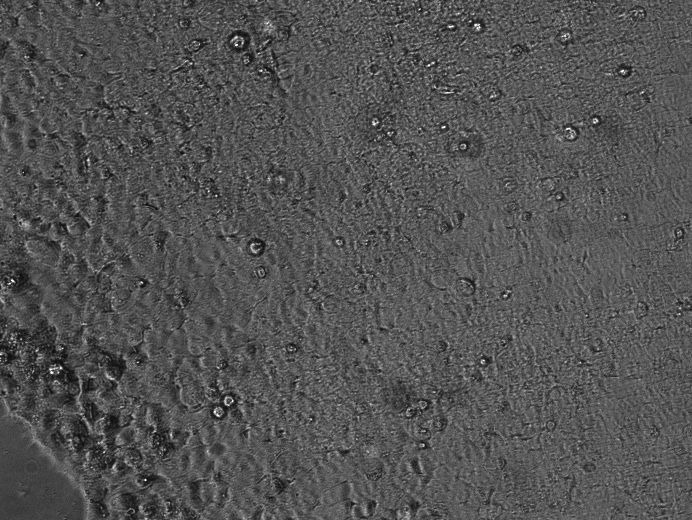

Supplement: Supplementary file 11 — Source data Fig. 1 [file 44318_2024_331_MOESM11_ESM.zip › SD figure 1/1A/hnRNPM-GFP/Aufnahme-3008-hnRNPM-GFP +pBluescript trans.tif]

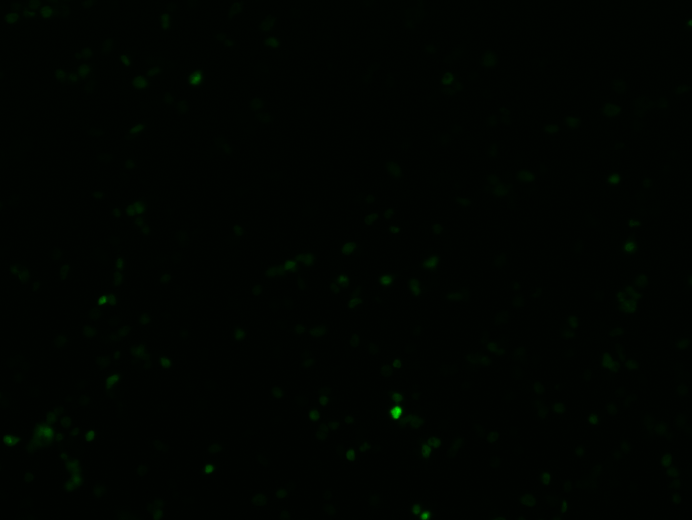

Supplement: Supplementary file 11 — Source data Fig. 1 [file 44318_2024_331_MOESM11_ESM.zip › SD figure 1/1A/hnRNPM-GFP/Aufnahme-3008-hnRNPM-GFP PV-FLAG.tif]

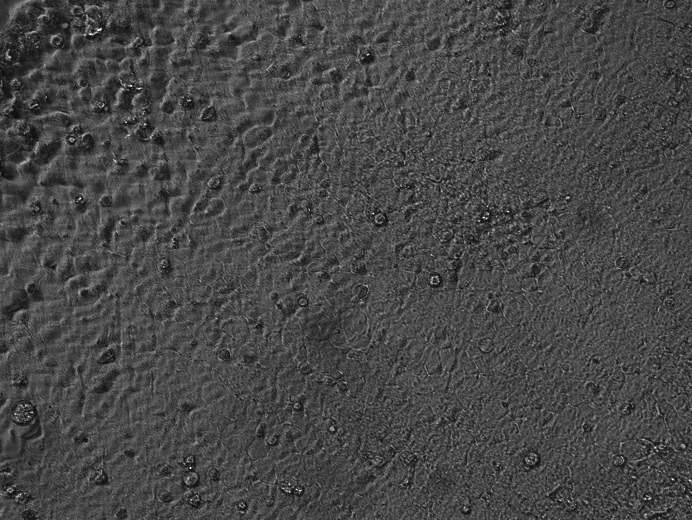

Supplement: Supplementary file 11 — Source data Fig. 1 [file 44318_2024_331_MOESM11_ESM.zip › SD figure 1/1A/GFP/Aufnahme-3008-hnRNPM-GFP CVB3-FLAG trans.tif]

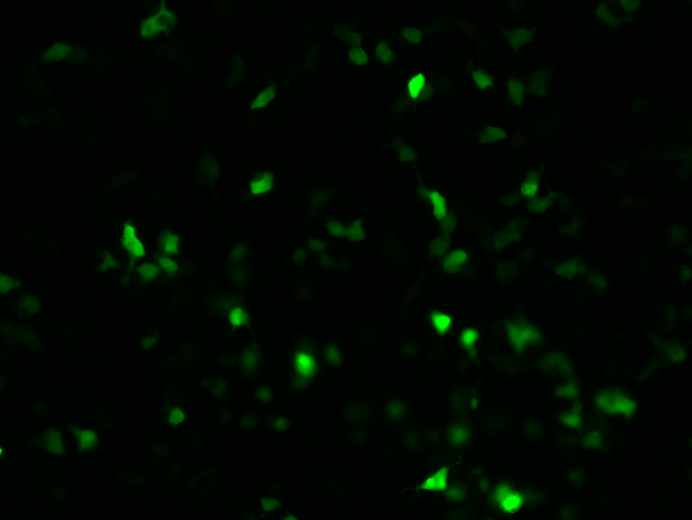

Supplement: Supplementary file 11 — Source data Fig. 1 [file 44318_2024_331_MOESM11_ESM.zip › SD figure 1/1A/GFP/Aufnahme-3008-hnRNPM-GFP +pBluescript-1.tif]

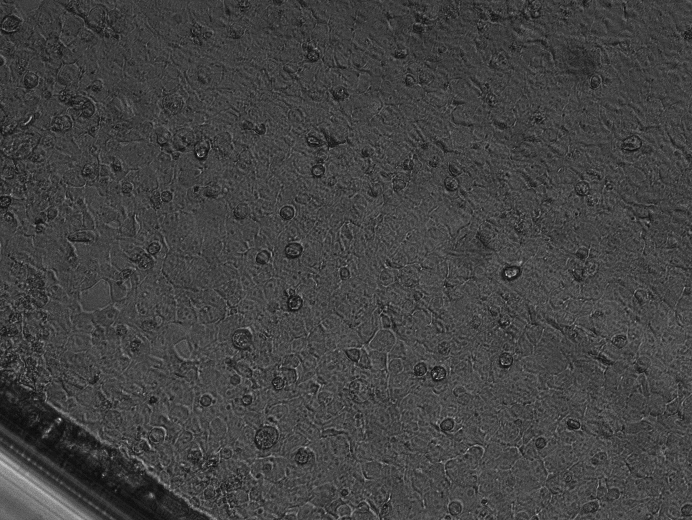

Supplement: Supplementary file 11 — Source data Fig. 1 [file 44318_2024_331_MOESM11_ESM.zip › SD figure 1/1A/GFP/Aufnahme-3008-hnRNPM-GFP PV-FLAG trans.tif]

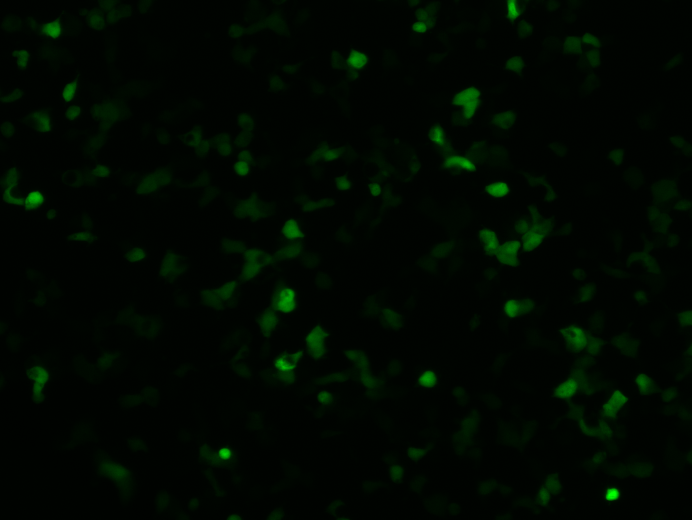

Supplement: Supplementary file 11 — Source data Fig. 1 [file 44318_2024_331_MOESM11_ESM.zip › SD figure 1/1A/GFP/Aufnahme-3008-hnRNPM-GFP PV-FLAG-1.tif]

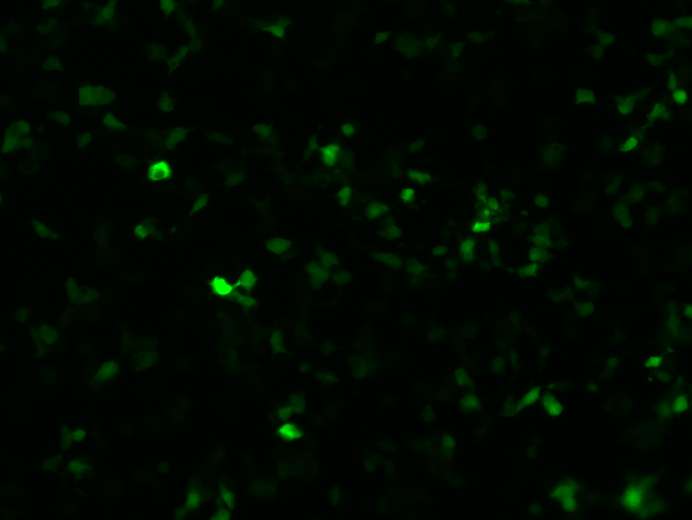

Supplement: Supplementary file 11 — Source data Fig. 1 [file 44318_2024_331_MOESM11_ESM.zip › SD figure 1/1A/GFP/Aufnahme-3008-hnRNPM-GFP CVB3-FLAG-1.tif]

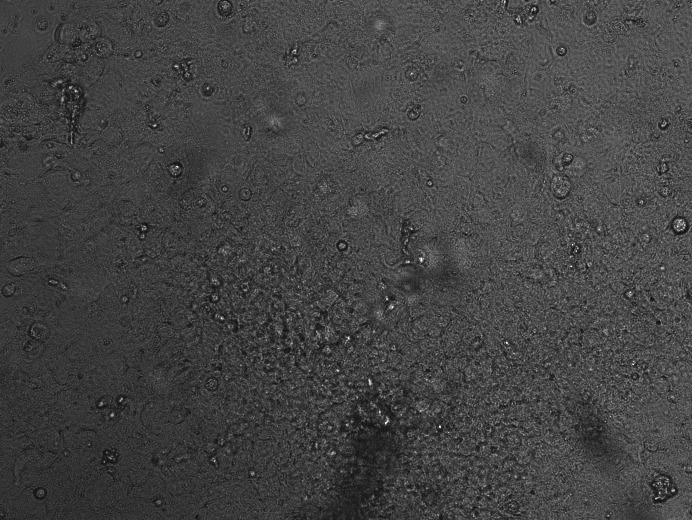

Supplement: Supplementary file 11 — Source data Fig. 1 [file 44318_2024_331_MOESM11_ESM.zip › SD figure 1/1A/GFP/Aufnahme-3008-hnRNPM-GFP +pBluescript trans.tif]

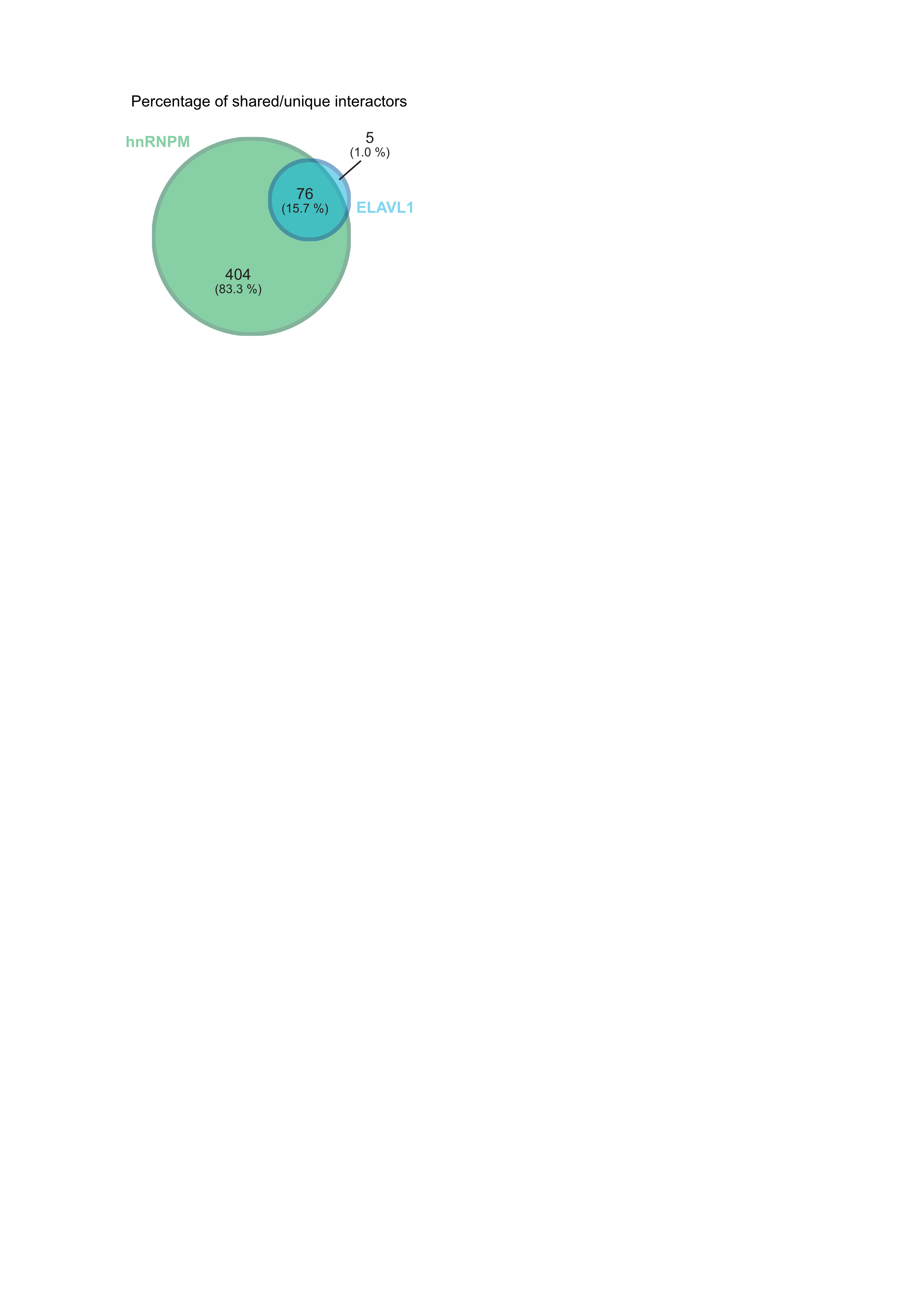

Supplement: Supplementary file 12 — Source data Fig. 3 [file 44318_2024_331_MOESM12_ESM.zip › SD figure 3/3E/3E.tiff]

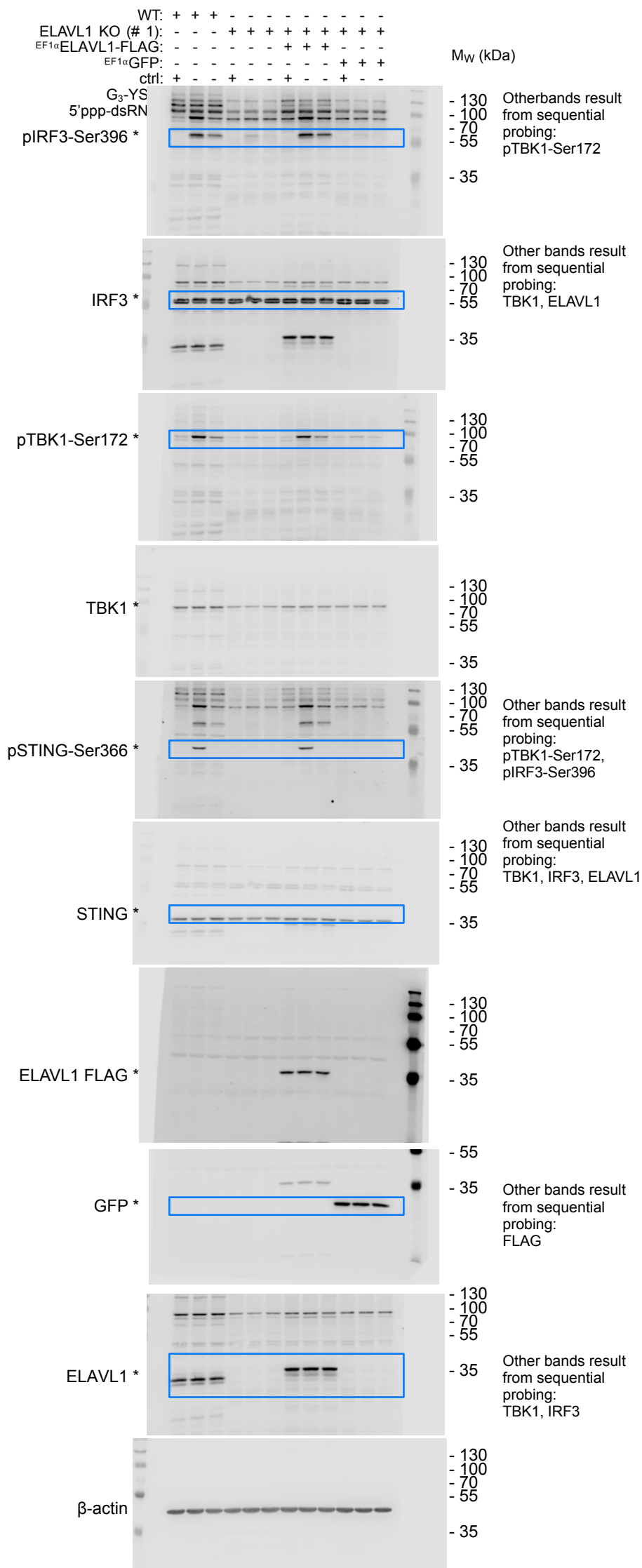

Supplement: Supplementary file 13 — Source data Fig. 4 [file 44318_2024_331_MOESM13_ESM.zip › SD figure 4/4J/4J.pdf]

Fig. 5 A (1/2)

IP: GFP

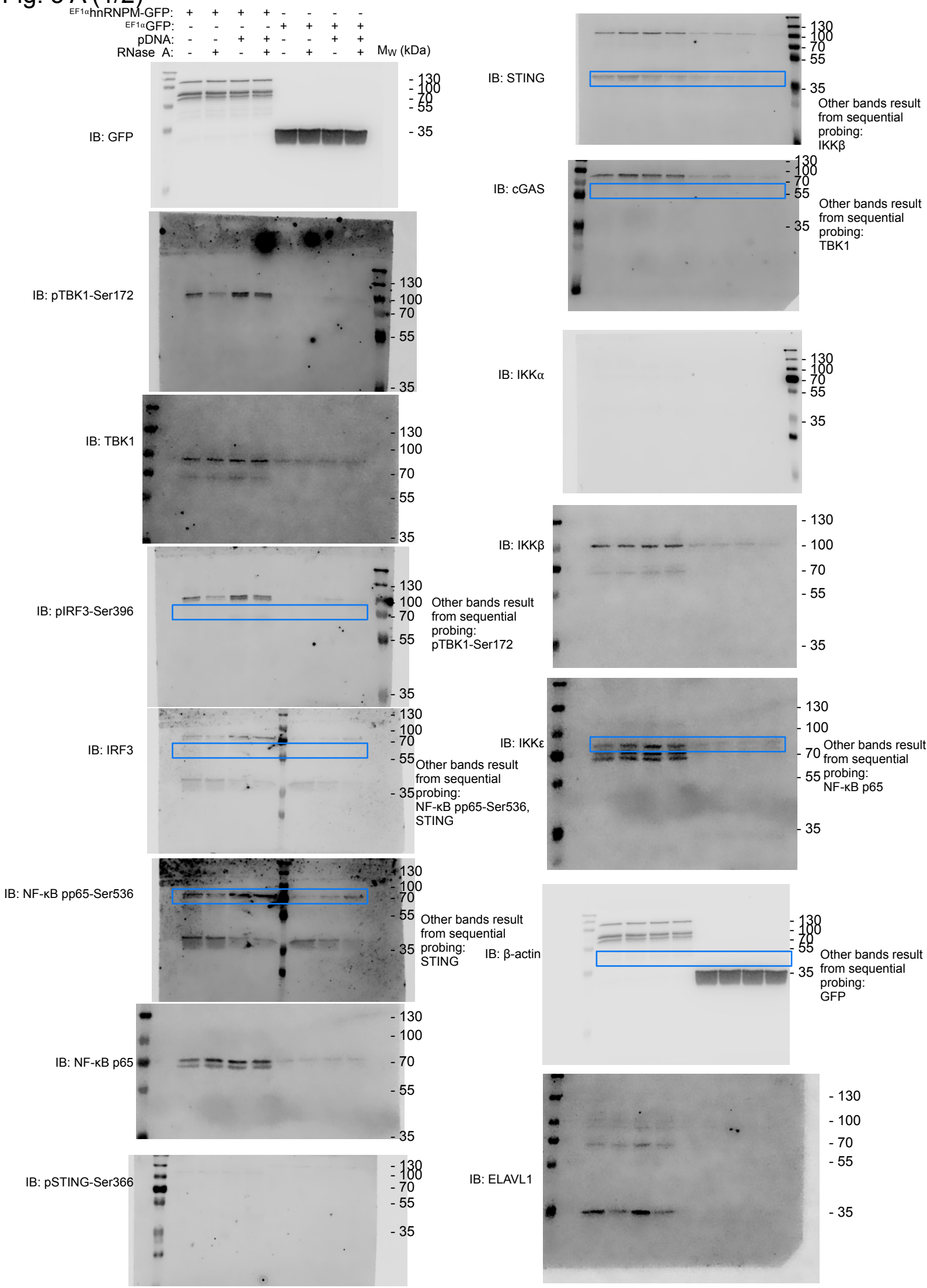

Supplement: Supplementary file 14 — Source data Fig. 5 [file 44318_2024_331_MOESM14_ESM.zip › SD figure 5/5A/5A_1.pdf]

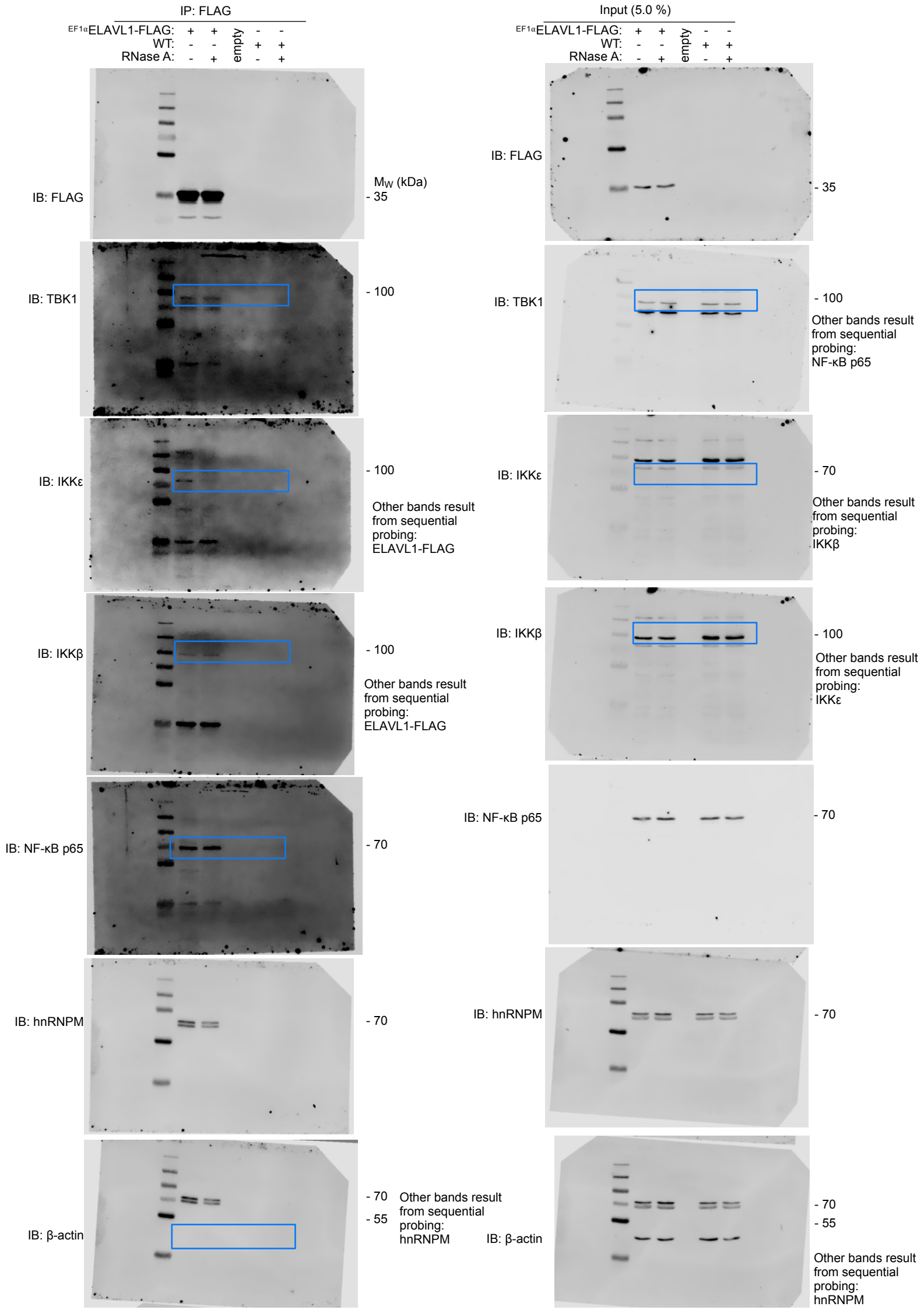

Supplement: Supplementary file 14 — Source data Fig. 5 [file 44318_2024_331_MOESM14_ESM.zip › SD figure 5/5B/5B.pdf]

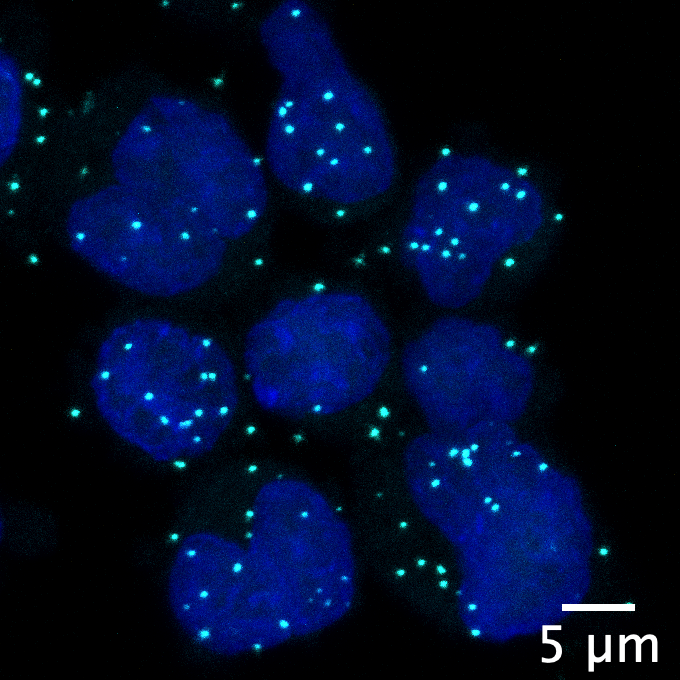

Supplement: Supplementary file 14 — Source data Fig. 5 [file 44318_2024_331_MOESM14_ESM.zip › SD figure 5/5C/ctrl/Composite (RGB).tif]

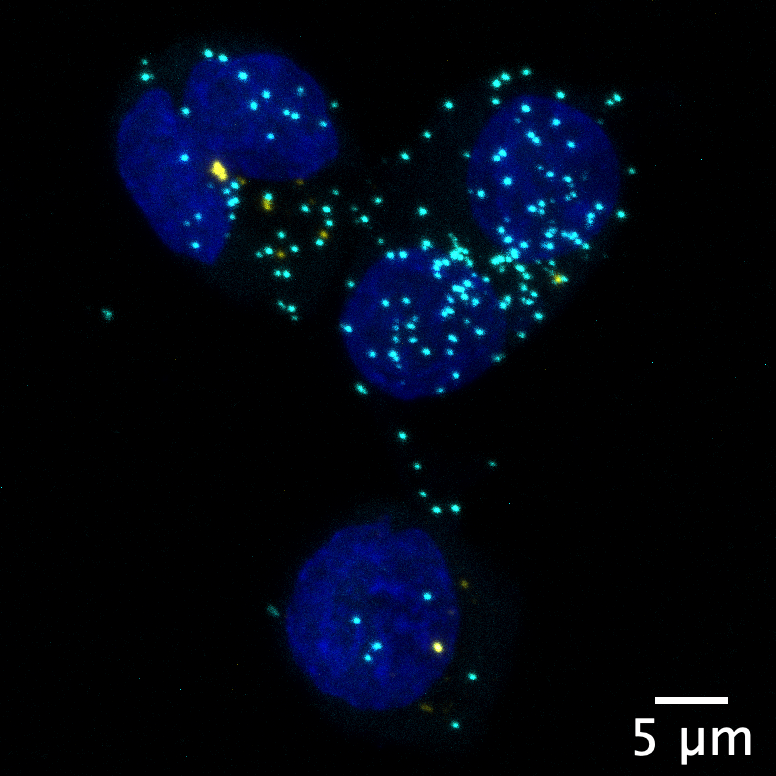

Supplement: Supplementary file 14 — Source data Fig. 5 [file 44318_2024_331_MOESM14_ESM.zip › SD figure 5/5C/G3-YSD/Composite (RGB).tif]

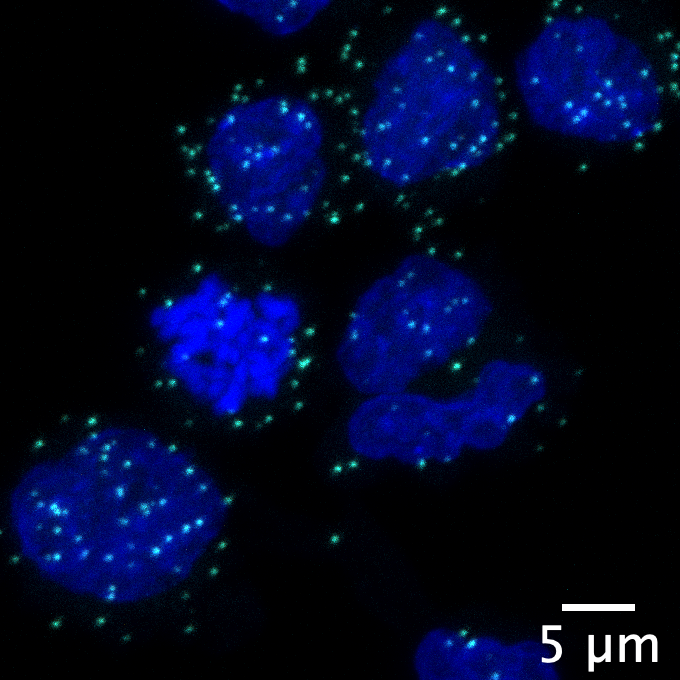

Supplement: Supplementary file 14 — Source data Fig. 5 [file 44318_2024_331_MOESM14_ESM.zip › SD figure 5/5D/ctrl/Composite (RGB).tif]

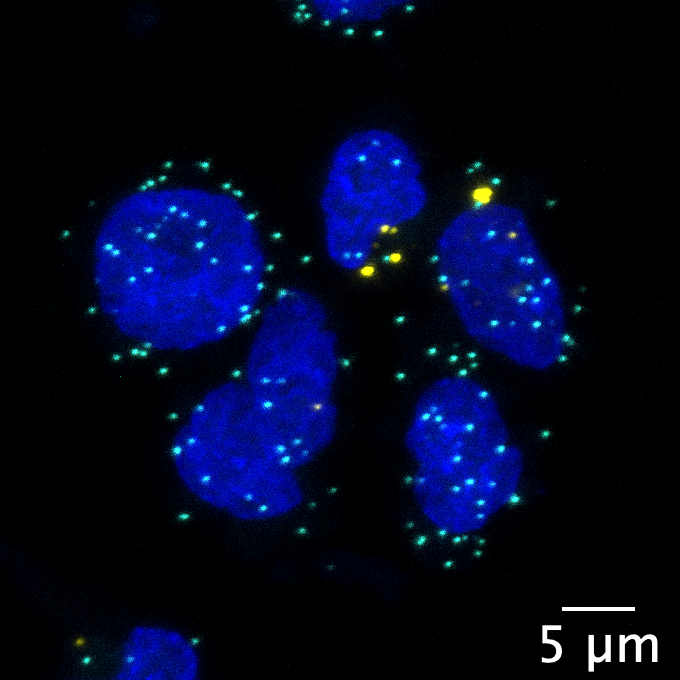

Supplement: Supplementary file 14 — Source data Fig. 5 [file 44318_2024_331_MOESM14_ESM.zip › SD figure 5/5D/G3-YSD/NEW___MAX_200814 PLA ELAVL1-GFP-pTBK1.lif - PLA ELAVL1-pTBK1 G3 5.tif (RGB)_scale bar.tif]

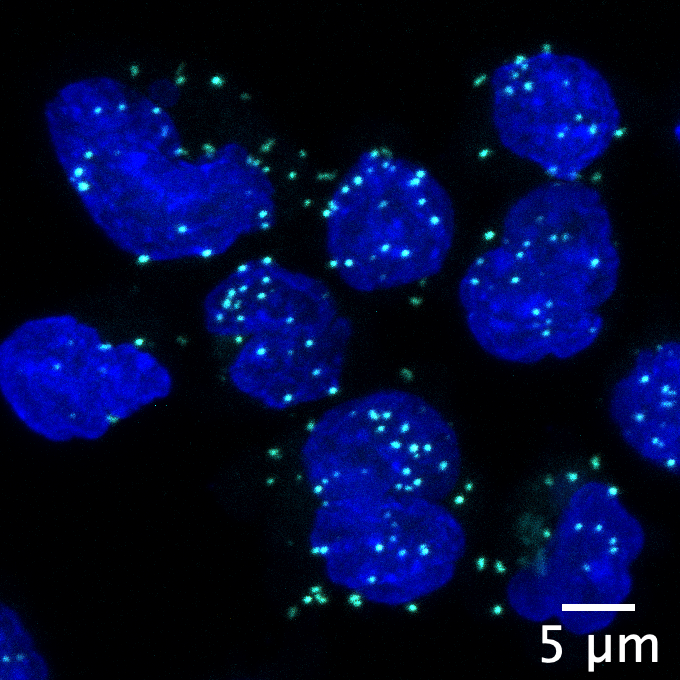

Supplement: Supplementary file 14 — Source data Fig. 5 [file 44318_2024_331_MOESM14_ESM.zip › SD figure 5/5E/ctrl/Composite (RGB).tif]

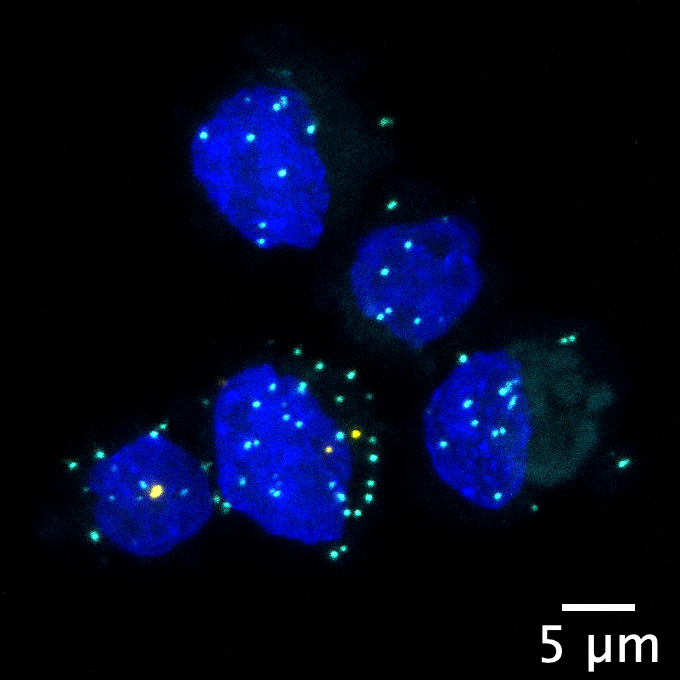

Supplement: Supplementary file 14 — Source data Fig. 5 [file 44318_2024_331_MOESM14_ESM.zip › SD figure 5/5E/G3-YSD/Composite (RGB).tif]

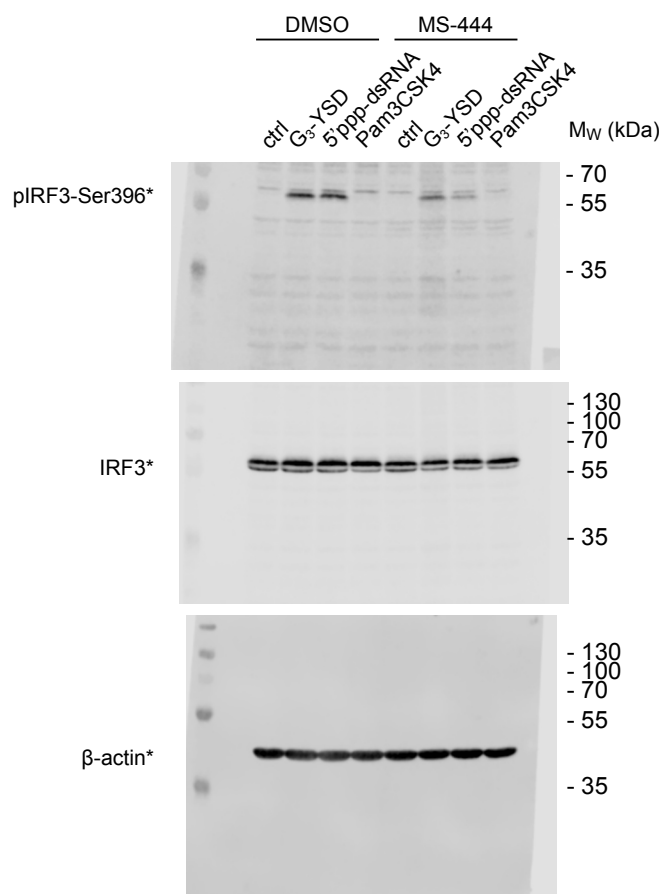

Supplement: Supplementary file 15 — Source data Fig. 6 [file 44318_2024_331_MOESM15_ESM.zip › SD figure 6/6C/6C.pdf]
