# Supplementary material for: RNA-binding proteins hnRNPM and ELAVL1 promote type-I interferon induction downstream of the nucleic acid sensors cGAS and RIG-I
Source: EMBO J. 2024 Dec 20;44(3):824–53. doi: 10.1038/s44318-024-00331-x (PMC11791083; doi:10.1038/s44318-024-00331-x)
Supplement: Supplementary file 13 — Source data Fig. 4 [file 44318_2024_331_MOESM13_ESM.zip › SD figure 4/4K/4K.pdf]

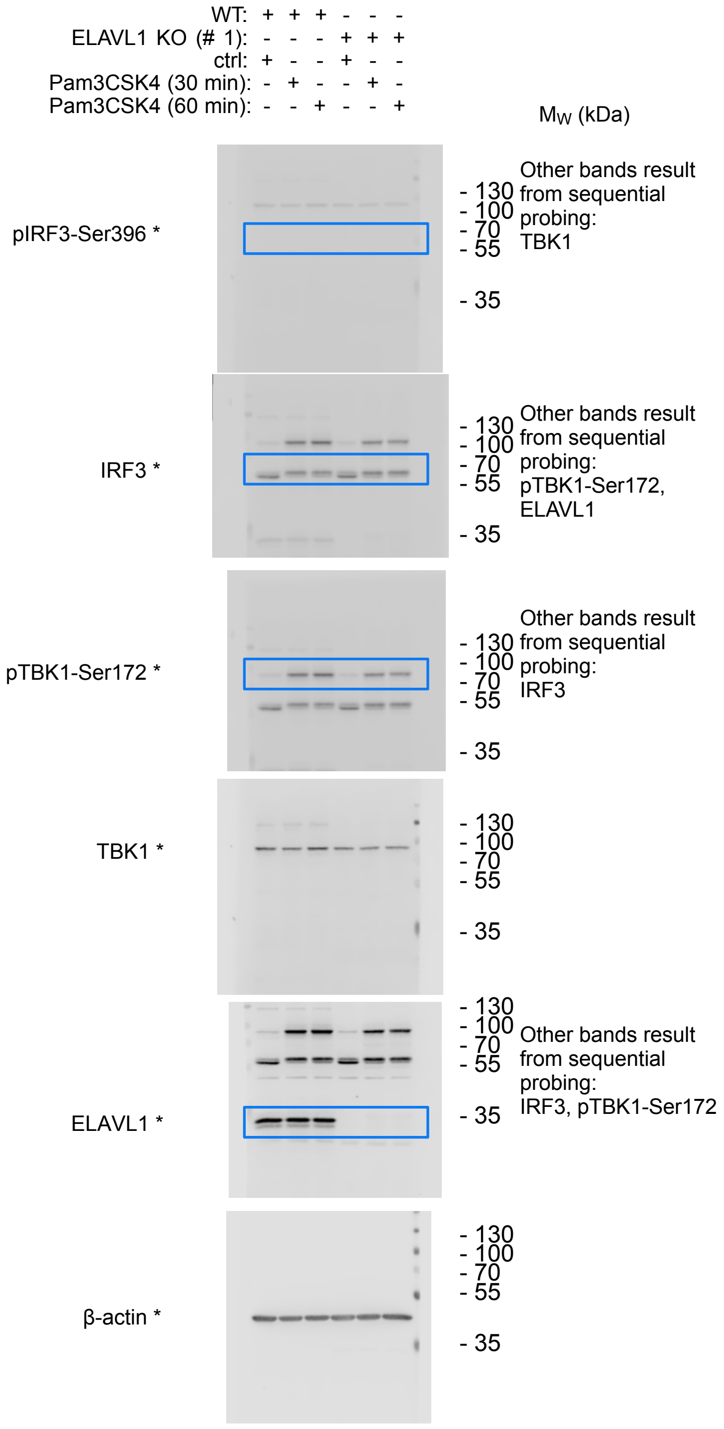
WT: + + + - - -
ELAVL1 KO (# 1): - - - + + +
ctrl: + - - + - -
Pam3CSK4 (30 min): - + - - + -
Pam3CSK4 (60 min): - - + - - +
MW (kDa)
pIRF3-Ser396 *
- 130
- 100
- 70
- 55
- 35
Other bands result from sequential probing: TBK1
IRF3 *
- 130
- 100
- 70
- 55
- 35
Other bands result from sequential probing: pTBK1-Ser172, ELAVL1
pTBK1-Ser172 *
- 130
- 100
- 70
- 55
- 35
Other bands result from sequential probing: IRF3
TBK1 *
- 130
- 100
- 70
- 55
- 35
ELAVL1 *
- 130
- 100
- 70
- 55
- 35
Other bands result from sequential probing: IRF3, pTBK1-Ser172
β-actin *
- 130
- 100
- 70
- 55
- 35
