# Supplementary material for: RNA-binding proteins hnRNPM and ELAVL1 promote type-I interferon induction downstream of the nucleic acid sensors cGAS and RIG-I
Source: EMBO J. 2024 Dec 20;44(3):824–53. doi: 10.1038/s44318-024-00331-x (PMC11791083; doi:10.1038/s44318-024-00331-x)
Supplement: Supplementary file 13 — Source data Fig. 4 [file 44318_2024_331_MOESM13_ESM.zip › SD figure 4/4A/4A.pdf]

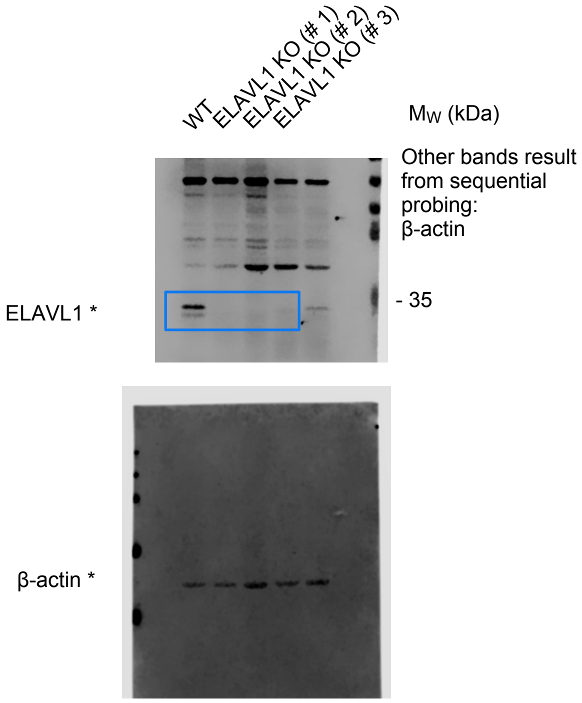
WT
ELAVL1 KO (# 1)
ELAVL1 KO (# 2)
ELAVL1 KO (# 3)
M_W (kDa)
Other bands result from sequential probing: β-actin
- 35
ELAVL1 *
β-actin *
