# Supplementary material for: RNA-binding proteins hnRNPM and ELAVL1 promote type-I interferon induction downstream of the nucleic acid sensors cGAS and RIG-I
Source: EMBO J. 2024 Dec 20;44(3):824–53. doi: 10.1038/s44318-024-00331-x (PMC11791083; doi:10.1038/s44318-024-00331-x)
Supplement: Supplementary file 14 — Source data Fig. 5 [file 44318_2024_331_MOESM14_ESM.zip › SD figure 5/5A/5A_2.pdf]

Fig. 5 A (2/2)

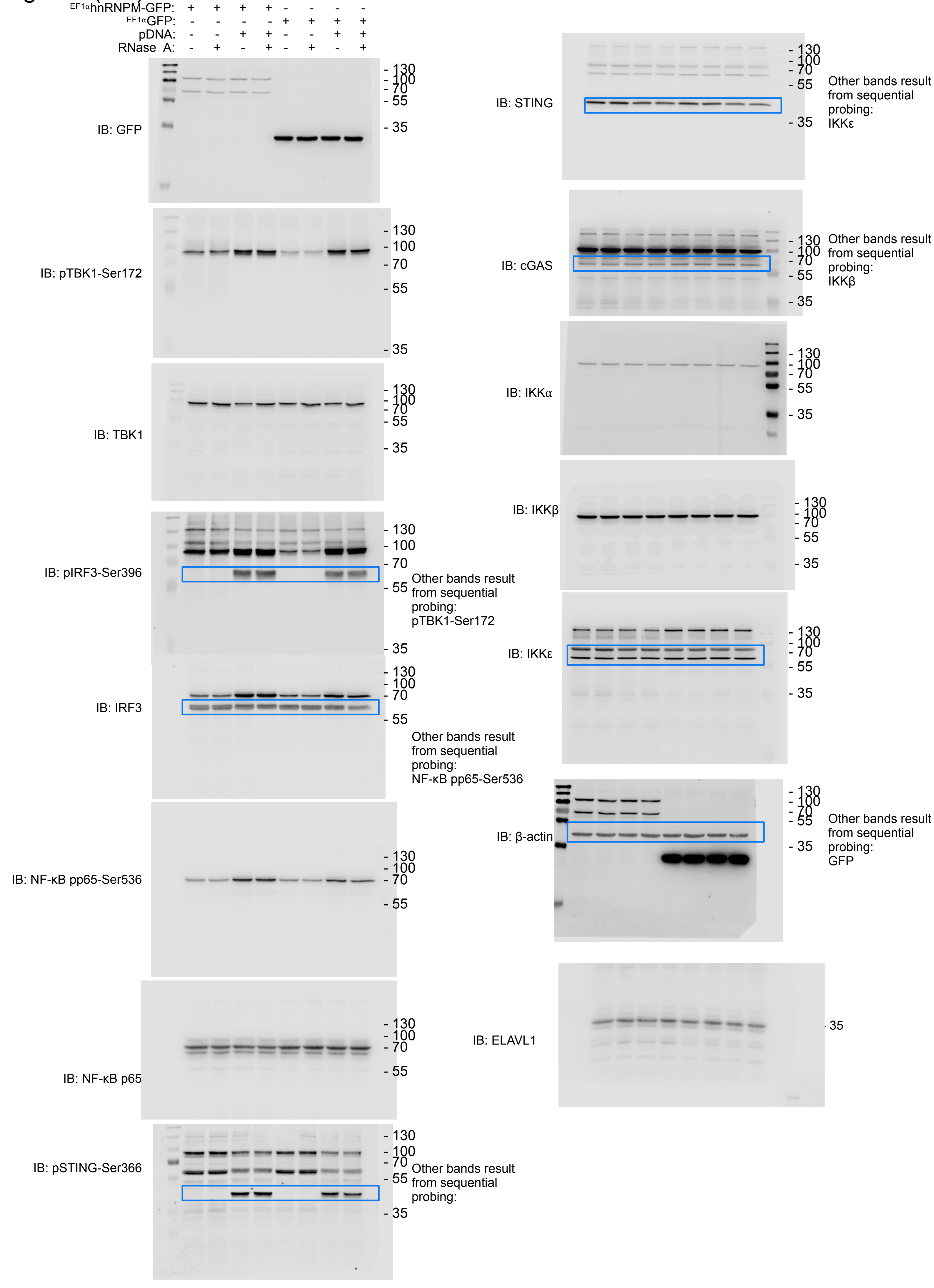
Input (5.0 %)
EF1α hnRNPM-GFP: + + + + - - - -
EF1α GFP: - - - - + + + +
pDNA: - - + + - - + +
RNase A: - + - + - + - +
IB: GFP
IB: pTBK1-Ser172
IB: TBK1
IB: pIRF3-Ser396
Other bands result from sequential probing: pTBK1-Ser172
IB: IRF3
Other bands result from sequential probing: NF-κB pp65-Ser536
IB: NF-κB pp65-Ser536
IB: NF-κB p65
IB: pSTING-Ser366
Other bands result from sequential probing:
IB: STING
Other bands result from sequential probing: IKKε
IB: cGAS
Other bands result from sequential probing: IKKβ
IB: IKKα
IB: IKKβ
IB: IKKε
IB: β-actin
Other bands result from sequential probing: GFP
IB: ELAVL1
130
100
70
55
35
