# Supplementary material for: RNA-binding proteins hnRNPM and ELAVL1 promote type-I interferon induction downstream of the nucleic acid sensors cGAS and RIG-I
Source: EMBO J. 2024 Dec 20;44(3):824–53. doi: 10.1038/s44318-024-00331-x (PMC11791083; doi:10.1038/s44318-024-00331-x)
Supplement: Supplementary file 16 — Expanded View Figures [file 44318_2024_331_MOESM16_ESM.pdf]

Expanded View Figures

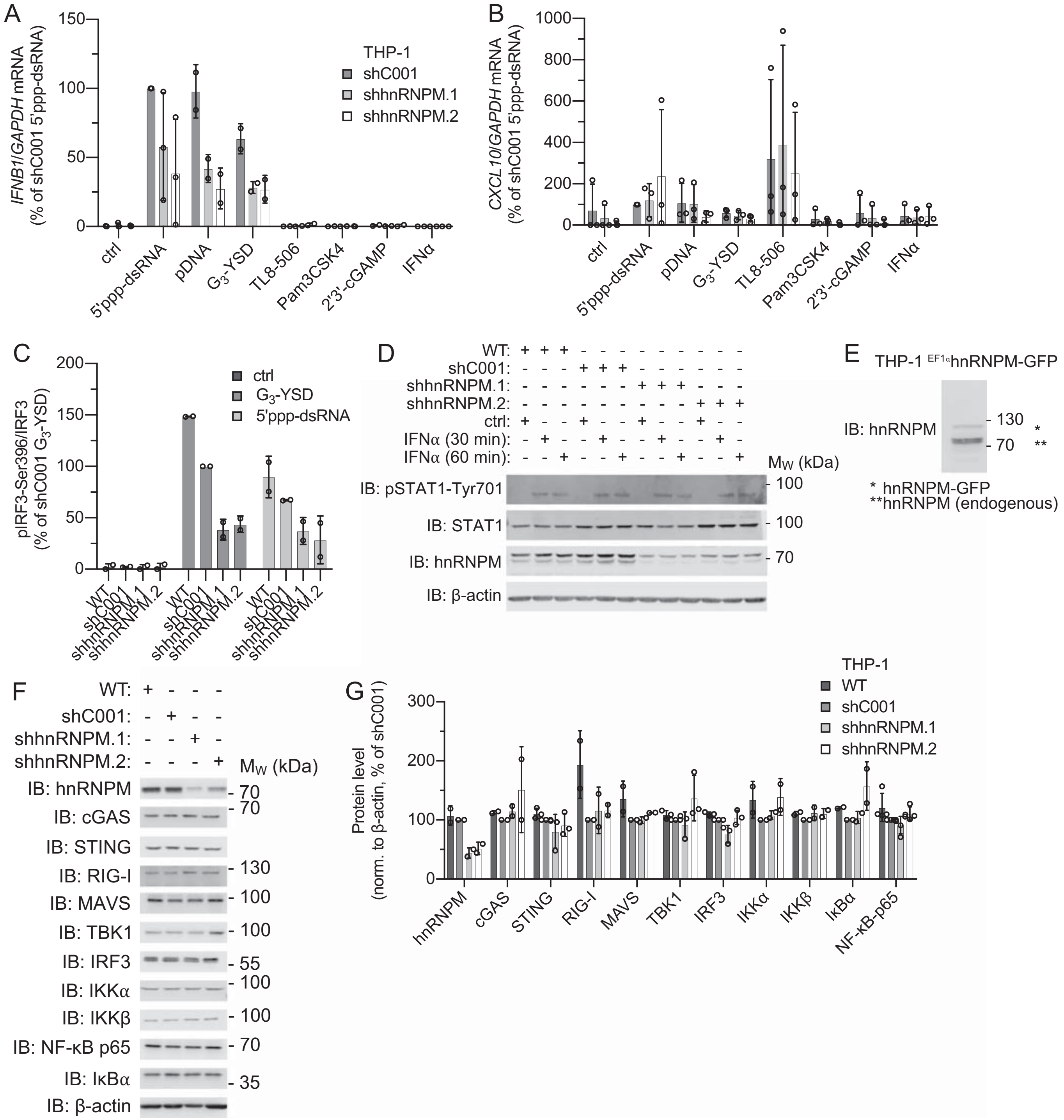

Figure EV1. hnRNPM functions upstream of IFNAR.

(**A**) Expression of *IFNB1* mRNA in THP-1 cells expressing control shRNA (shC001) or hnRNPM-specific shRNAs (shhnRNPM.1, shhnRNPM.2) 6 h after stimulation of RIG-I with 5′ppp-dsRNA (0.1 µg/ml), of cGAS with pDNA (0.1 µg/ml) or G_3-YSD (0.5 µg/ml), of TLR8 with TL8-506 (1.0 µg/ml), of TLR1/2 with Pam3CSK4 (0.5 µg/ml), of STING with 2′3′-cGAMP (10 µg/ml), or of IFNAR with IFNα (1000 U/ml). ctrl, non-stimulated (mean ± SD; stimuli from left to right: $n = 3, 3, 2, 2, 2, 2, 2, 2$ independent experiments). (**B**) Expression of *CXCL10* mRNA in the cells depicted in (**A**) 6 h after stimulation with the indicated stimuli (mean ± SD; stimuli from left to right: $n = 3, 3, 3, 3, 3, 3, 3, 3$ independent experiments). (**C**) Quantification of pIRF3-Ser396 levels shown in Fig. 1I (mean ± SD; stimuli from left to right: $n = 2, 2, 2$ independent experiments). (**D**) Immunoblot analysis of pSTAT1-Tyr701 induction in THP-1 WT and cells expressing control shRNA (shC001) or hnRNPM-specific shRNAs (shhnRNPM.1, shhnRNPM.2) after stimulation with IFNα (1000 U/ml). ctrl, non-stimulated. One representative experiment of two independent experiments is shown. (**E**) Immunoblot analysis of THP-1 cells expressing hnRNPM-GFP using an hnRNPM-specific antibody. (**F**) Immunoblot analysis of THP-1 WT and cells expressing control shRNA (shC001) or hnRNPM-specific shRNAs (shhnRNPM.1, shhnRNPM.2) using the indicated antibodies. One representative experiment of two independent experiments is shown. (**G**) Quantification of immunoblot data shown in Fig. EV1F (mean ± SD; proteins from left to right: $n = 2, 2, 3, 2, 2, 3, 3, 2, 2, 2, 4$ independent experiments).

 © The Author(s)

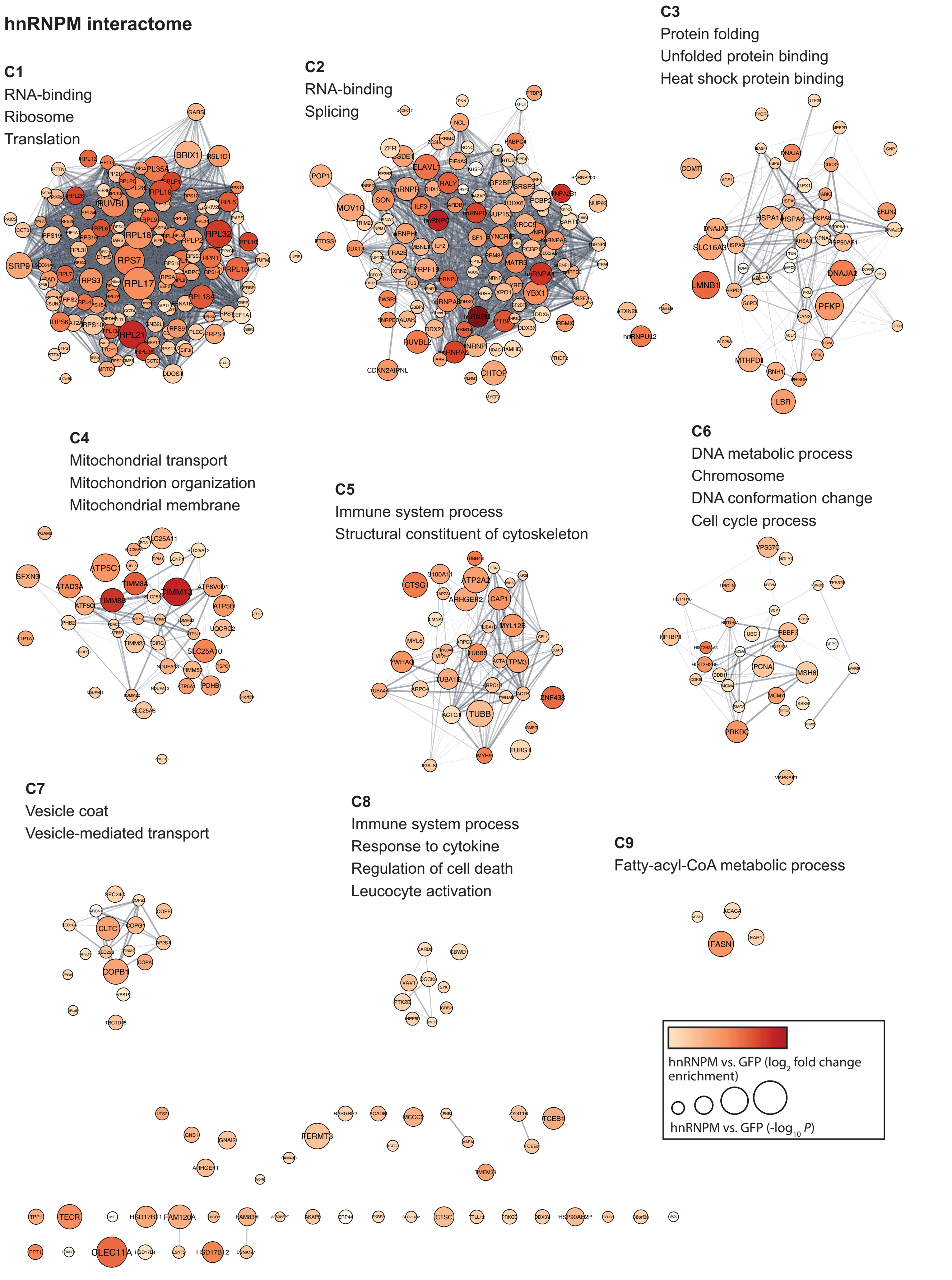
hnRNPM interactome
C1
RNA-binding
Ribosome
Translation
C2
RNA-binding
Splicing
C3
Protein folding
Unfolded protein binding
Heat shock protein binding
C4
Mitochondrial transport
Mitochondrion organization
Mitochondrial membrane
C5
Immune system process
Structural constituent of cytoskeleton
C6
DNA metabolic process
Chromosome
DNA conformation change
Cell cycle process
C7
Vesicle coat
Vesicle-mediated transport
C8
Immune system process
Response to cytokine
Regulation of cell death
Leucocyte activation
C9
Fatty-acyl-CoA metabolic process
hnRNPM vs. GFP (log2 fold change enrichment)
hnRNPM vs. GFP (-log10 P)

Figure EV2. hnRNPM interacts with proteins connected to immune system processes.

hnRNPM-GFP and GFP were immunoprecipitated from lysates of non-stimulated THP-1 cells. Differential interactors of hnRNPM were analyzed by STRING enrichment and annotated with GO terms enriched among hnRNPM interactors. hnRNPM interactors not annotated with these GO terms are shown at the bottom. cluster (C) 1–9. The statistical tests used are described in detail in the methods.

 © The Author(s)

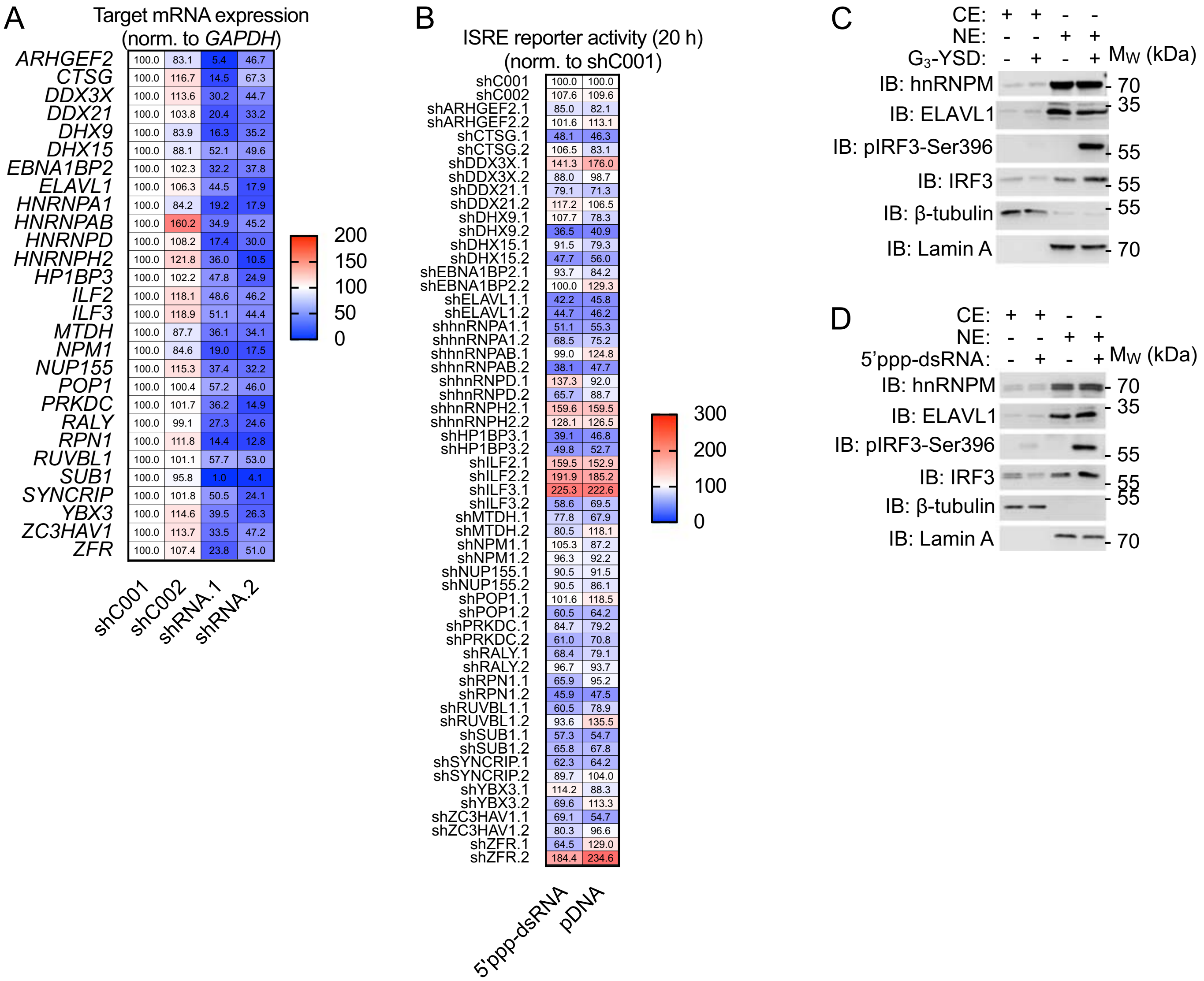

Figure EV3. Functional RNAi screen of hnRNPM interactors.

(**A**) mRNA expression of the indicated targets (y-axis) in THP-1 cells expressing control shRNAs (shC001, shC002) or target-specific shRNAs (shRNA.1, shRNA.2). Target mRNA expression was normalized to *GAPDH* mRNA and then normalized to shC001-expressing cells. Data of two or more independent experiments are shown. (**B**) ISRE reporter activation in the cells depicted in (A) 20 h after stimulation with 5′ppp-dsRNA (0.1 µg/ml) or pDNA (0.1 µg/ml). Luciferase signals were normalized to shC001-expressing cells of the respective condition. Data of two or more independent experiments are shown. (**C**) Nuclear extracts (NE) and cytoplasmic extracts (CE) were prepared from non-stimulated THP-1 cells or from cells stimulated with G_3-YSD (0.5 µg/ml) for 3 h and analyzed by immunoblotting with the indicated antibodies. One representative experiment of two independent experiments is shown. (**D**) NE and CE were prepared from non-stimulated THP-1 cells or from cells stimulated with 5′ppp-dsRNA (0.1 µg/ml) for 3 h and analyzed by immunoblotting with the indicated antibodies. One representative experiment of two independent experiments is shown.

© The Author(s)

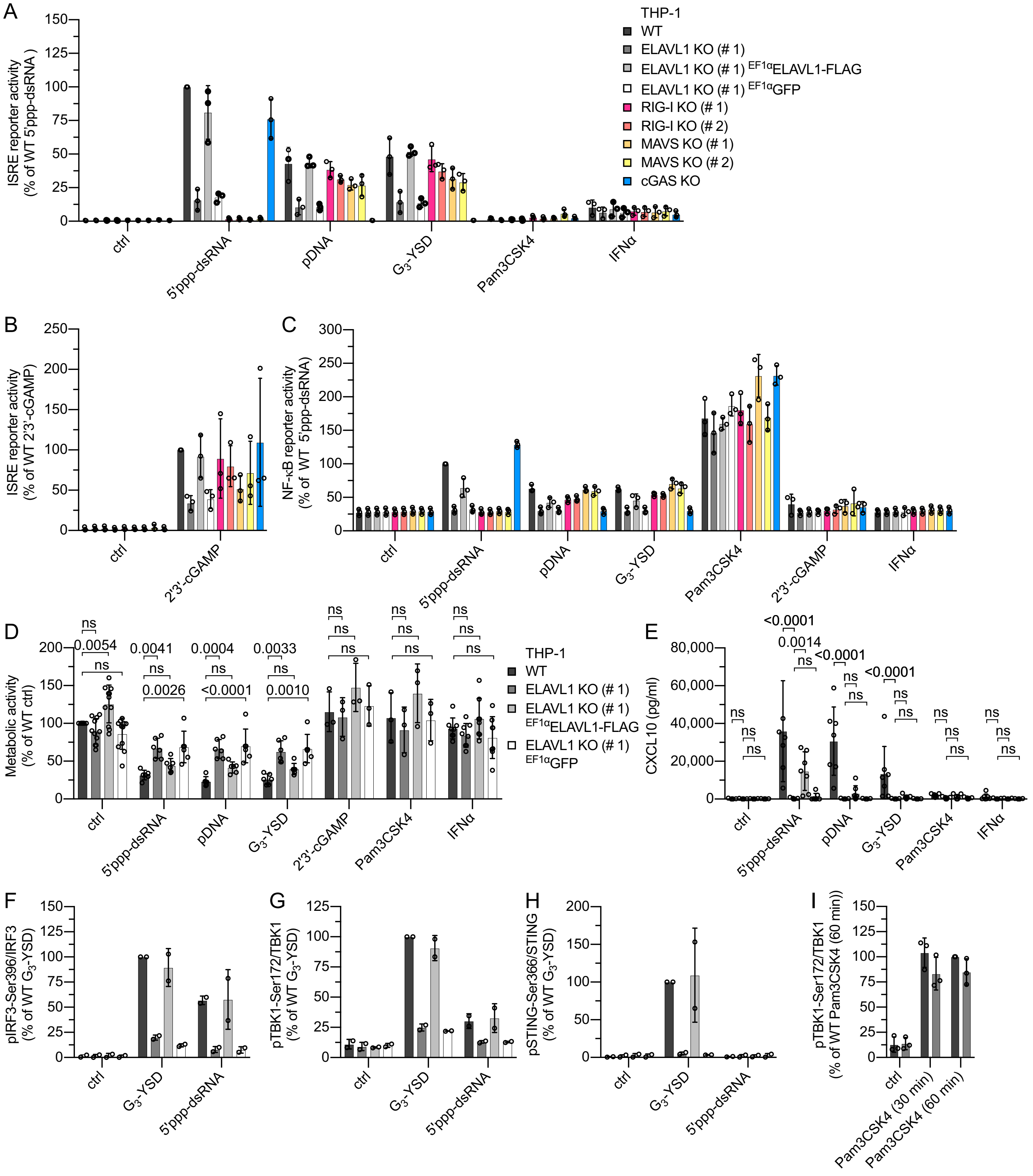
A
ISRE reporter activity
(% of WT 5'ppp-dsRNA)
THP-1
WT
ELAVL1 KO (# 1)
ELAVL1 KO (# 1) EF1αELAVL1-FLAG
ELAVL1 KO (# 1) EF1αGFP
RIG-I KO (# 1)
RIG-I KO (# 2)
MAVS KO (# 1)
MAVS KO (# 2)
cGAS KO
ctrl
5'ppp-dsRNA
pDNA
G3-YSD
Pam3CSK4
IFNα
B
ISRE reporter activity
(% of WT 2'3'-cGAMP)
2'3'-cGAMP
C
NF-κB reporter activity
(% of WT 5'ppp-dsRNA)
D
Metabolic activity
(% of WT ctrl)
ns
0.0054
0.0041
0.0026
0.0004
<0.0001
0.0033
0.0010
ELAVL1 KO (# 1) EF1αELAVL1-FLAG
ELAVL1 KO (# 1) EF1αGFP
E
CXCL10 (pg/ml)
<0.0001
0.0014
F
pIRF3-Ser396/IRF3
(% of WT G3-YSD)
G
pTBK1-Ser172/TBK1
(% of WT G3-YSD)
H
pSTING-Ser366/STING
(% of WT G3-YSD)
I
pTBK1-Ser172/TBK1
(% of WT Pam3CSK4 (60 min))
Pam3CSK4 (30 min)
Pam3CSK4 (60 min)

© The Author(s)

Figure EV4. ELAVL1 is a potent positive regulator of cGAS and RIG-I signaling.

(**A**) ISRE reporter activation in THP-1 WT, ELAVL1 KO (#1), ELAVL1 KO (#1) expressing ELAVL1-FLAG or GFP, RIG-I KO (clones #1–2), MAVS KO (clones #1–2), and cGAS KO cells 20 h after stimulation with 5′ppp-dsRNA (0.1 µg/ml), pDNA (0.1 µg/ml), G_3-YSD (0.5 µg/ml), Pam3CSK4 (0.5 µg/ml), or IFNα (5000 U/ml) (mean ± SD; stimuli from left to right: *n* = 3, 3, 3, 3, 3, 3 independent experiments). ctrl, non-stimulated. (**B**) ISRE reporter activation in the cells depicted in (**A**) 20 h after challenge with 2′3′-cGAMP (10 µg/ml) (mean ± SD; stimuli from left to right: *n* = 3, 3 independent experiments). ctrl, non-stimulated. (**C**) NF-κB reporter activation in the cells depicted in (A) 20 h after challenge with the indicated stimuli (mean ± SD; stimuli from left to right: *n* = 3, 3, 3, 3, 3, 3, 3 independent experiments). (**D**) MTT assay of THP-1 WT, ELAVL1 KO (#1), and ELAVL1 KO (#1) expressing ELAVL1-FLAG or GFP 20 h after stimulation with 5′ppp-dsRNA (0.1 µg/ml), pDNA (0.1 µg/ml), G_3-YSD (0.5 µg/ml), 2′3′-cGAMP (10 µg/ml), Pam3CSK4 (0.5 µg/ml), or IFNα (1000 U/ml). ctrl, non-stimulated (stimuli from left to right: *n* = 11, 6, 6, 6, 3, 3, 8 independent experiments). (**E**) CXCL10 ELISA with supernatants of the cells depicted in (**D**) collected 20 h after stimulation with 5′ppp-dsRNA (0.1 µg/ml), pDNA (0.1 µg/ml), G_3-YSD (0.5 µg/ml), Pam3CSK4 (0.5 µg/ml), or IFNα (1000 U/ml). ctrl, non-stimulated (stimuli from left to right: *n* = 7, 7, 7, 7, 7, 7 independent experiments). (**F**) Quantification of pIRF3-Ser396 levels shown in Fig. 4J (mean ± SD; stimuli from left to right: *n* = 2, 2, 2 independent experiments). (**G**) Quantification of pTBK1-Ser172 levels shown in Fig. 4J (mean ± SD; stimuli from left to right: *n* = 2, 2, 2 independent experiments). (**H**) Quantification of pSTING-Ser366 levels shown in Fig. 4J (mean ± SD; stimuli from left to right: *n* = 2, 2, 2 independent experiments). (**I**) Quantification of pTBK1-Ser172 levels shown in Fig. 4K (mean ± SD; stimuli from left to right: *n* = 3, 3, 3 independent experiments). For (**D**, **E**): mean ± SD, two-way ANOVA, Dunnett's multiple comparisons test. ns, *P* value > 0.05.

© The Author(s)

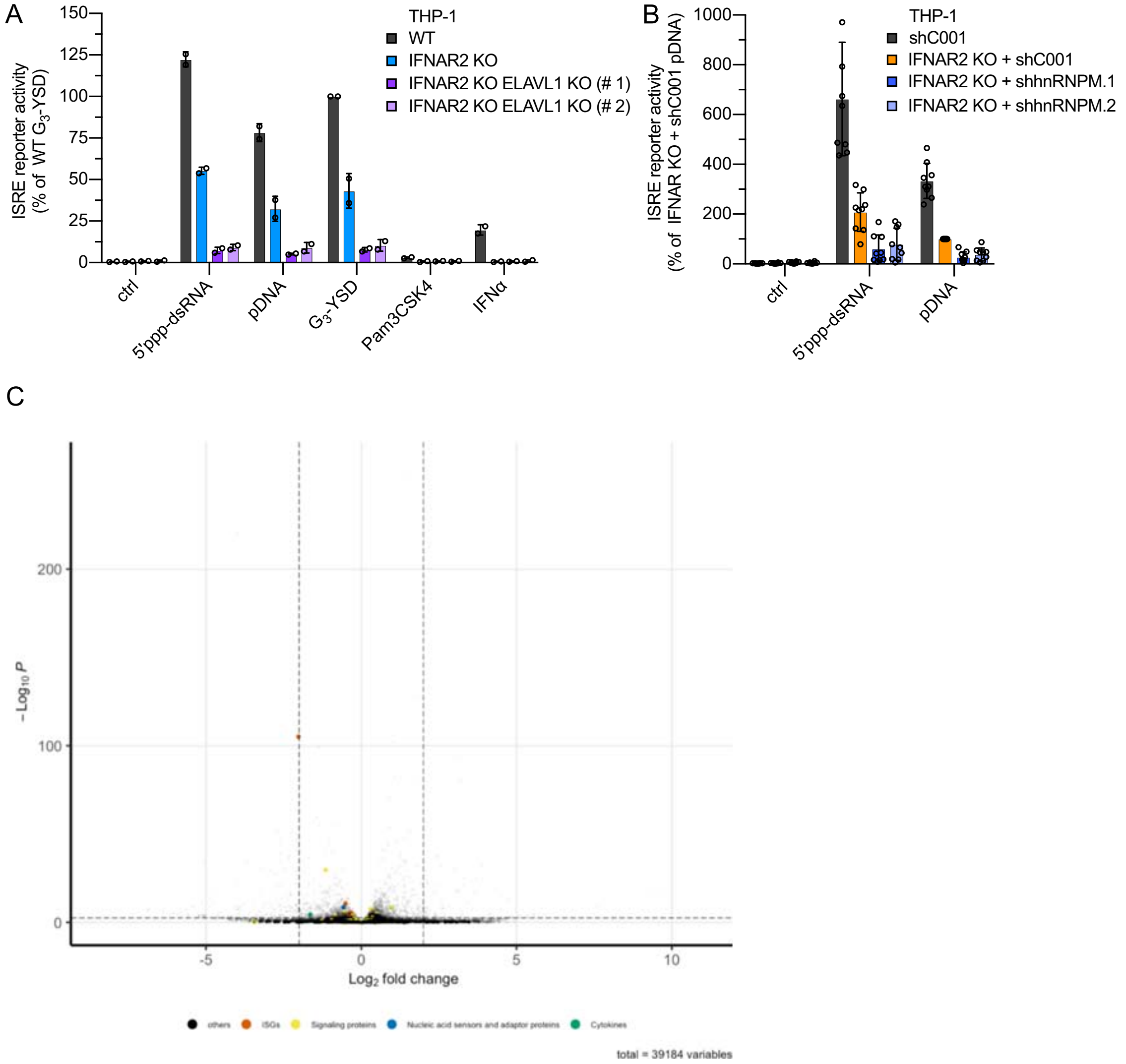

Figure EV5. ELAVL1 regulates signal transduction downstream of cGAS and RIG-I.

(**A**) ISRE reporter activation in THP-1 WT, IFNAR2 KO and IFNAR2/ELAVL1 double-KO cells (clones #1–2, ELAVL1 gRNA AN) 20 h after stimulation with 5′ppp-dsRNA (0.1 µg/ml), pDNA (0.1 µg/ml), G_3-YSD (0.5 µg/ml), Pam3CSK4 (0.5 µg/ml), or IFNα (5000 U/ml) (mean ± SD: stimuli from left to right: $n = 2, 2, 2, 2, 2, 2$ independent experiments). ctrl, non-stimulated. (**B**) ISRE reporter activation in shC001-expressing THP-1 cells and IFNAR2 KO cells expressing shC001, shhnRNPM.1, or shhnRNPM.2 20 h after stimulation with 5′ppp-dsRNA (0.1 µg/ml) or pDNA (0.1 µg/ml) (mean ± SD; stimuli from left to right: $n = 10, 9, 9$ independent experiments).). ctrl, non-stimulated. (**C**) 3′-mRNA sequencing of total RNA from IFNα-stimulated (1000 U/ml, 6 h) THP-1 ELAVL1 KO (#1) and WT cells. Wald test was used to identify differentially expressed genes, with *P* values adjusted for multiple testing using the Benjamini–Hochberg procedure. The Volcano plot correlates the gene expression ($\log_2$ fold change of ELAVL1 KO vs. WT cells) with the $-\log_{10}$ adjusted *P* value ($P_{adjusted}$). Significantly regulated genes were defined as $P_{adjusted} < 0.05$ and log2 fold change >2 or log2 fold change > -2 (nucleic acid sensors and adaptor proteins (blue): cGAS, DDX58, IFIH1, MAVS, TMEM173; signaling proteins (yellow): IKBIP, TBK1, CHUK, IKBKB, IKBKE, IRF1, IRF2, IRF3, IRF4, IRF5, IRF7, IRF9, TICAM1, MYD88, TRAF1, TRAF2, TRAF3, TRAF5, TRAF6, TRAF7, TRIM25, RNF135, HMGB1, TFAM, ZCCHC3, G3BP1, NONO, IFI16, TTLL4, TTLL6, IFI16, DDX60, DHX58, IFNAR1, IFNAR2; ISGs (red): IFIT1, IFIT2, IFIT3, MX1, IL6, TNFA, IFI44L, IFI16, OASL, OAS1, OAS2, OAS3; cytokines (green): IFNB1, IFNL1, CXCL10).

 © The Author(s)
